# Effects of Non‐Aspirin Nonsteroidal Anti‐Inflammatory Drugs on Acute Intracerebral Hemorrhage

**DOI:** 10.1002/acn3.70163

**Published:** 2025-08-15

**Authors:** Shin‐Joe Yeh, Sung‐Chun Tang, Li‐Kai Tsai, Jiann‐Shing Jeng

**Affiliations:** ^1^ Department of Neurology National Taiwan University Hospital Taipei Taiwan

**Keywords:** functional outcome, intracerebral hemorrhage, mortality, non‐aspirin nonsteroidal anti‐inflammatory drugs, operation

## Abstract

**Objective:**

Despite celecoxib, a cyclooxygenase‐2 inhibitor, promoting functional recovery from intracerebral hemorrhage (ICH) by reducing inflammation‐mediated perihematomal edema in rat models, the evidence of its effects on patient outcomes remains limited. As nonsteroidal anti‐inflammatory drugs (NSAIDs) alleviate inflammation by inhibiting cyclooxygenase‐2, this study aimed to assess the impact of non‐aspirin NSAIDs on ICH outcomes.

**Methods:**

Patients with acute ICH admitted to our hospital between January 2015 and December 2020 were prospectively enrolled and retrospectively categorized based on pre‐ or post‐ICH use of non‐aspirin NSAIDs. Outcomes were assessed using the modified Rankin Scale (mRS) score at 3 months, survival at 1 year, and mortality at long‐term follow‐up.

**Results:**

Among 976 patients with acute ICH, 2.0% and 15.0% were non‐aspirin NSAID users before and after ICH, respectively. Post‐ICH non‐aspirin NSAID use was associated with a reduced 1‐year mortality risk (adjusted odds ratio [aOR] 0.30, *p* = 0.001) and long‐term mortality risk (adjusted hazard ratio 0.56, *p* = 0.043), but not good functional outcomes (mRS 0–2) (aOR 0.98, *p* = 0.940). In the subgroup analyses, post‐ICH use might be linked to good functional outcomes in patients with lobar hemorrhage or in those without surgical intervention. Pre‐ICH non‐aspirin NSAID use was not associated with these outcomes in the overall population, but it might be linked to increased mortality in subgroups with lobar hemorrhage, cerebral amyloid angiopathy, hyperlipidemia, or without intraventricular hemorrhage.

**Interpretation:**

The post‐ICH use of non‐aspirin NSAIDs reduced mortality. Future studies are warranted to identify specific non‐aspirin NSAID regimens that can significantly improve the outcomes of patients with ICH.

## 1 Introduction

Intracerebral hemorrhage (ICH) is a devastating type of stroke with high mortality and disability; however, effective therapeutic options are limited [1]. The immediate injury caused by ICH is attributed to the mass effect of hematomas through direct mechanical disruption and compression [1]. Furthermore, perihematomal edema causes secondary injury, which is mainly induced by inflammatory reactions [2]. Although perihematomal edema is not consistently associated with clinical outcomes of ICH [3, 4, 5], it amplifies the overall mass effect of the hematomas and has been the focus of emerging therapies for ICH, particularly those aimed at modulating inflammatory responses [2].

Celecoxib, a selective cyclooxygenase‐2 (COX‐2) inhibitor, reduced perihematomal edema in both animal models and patients with ICH [6, 7, 8]. In addition, early use of celecoxib within 5 days of ICH improved the survival of patients [9]. However, the effects of celecoxib in promoting functional recovery from ICH were only observed in rat models, but not in humans [6, 7]. Therefore, evidence regarding the effects of celecoxib in improving outcomes in patients with ICH remains limited.

In addition to celecoxib, nonsteroidal anti‐inflammatory drugs (NSAIDs) inhibit COX‐2 to reduce inflammatory reactions [10]. We hypothesized that NSAIDs may improve ICH prognosis by reducing the inflammatory reactions around the hematoma. Aspirin is typically excluded from NSAID studies on ICH because of its additional antiplatelet effects, which might compromise hemostasis in acute ICH [11, 12, 13]. Till date, evidence regarding the impact of post‐ICH use of non‐aspirin NSAIDs on ICH outcomes remains limited. Furthermore, some findings indicate that the pre‐ICH use of non‐aspirin NSAIDs does not increase the risk of developing ICH [11, 13], or show any association with the functional outcomes of ICH [12]. However, whether the pre‐ICH use of these drugs affects ICH characteristics or mortality remains unclear. To address these issues, this study aimed to investigate the influence of non‐aspirin NSAID use, before or after ICH, on the characteristics and outcomes of ICH.

## 2 Methods

### 2.1 Participants

The data for this study were retrospectively retrieved from a prospective stroke registry that consecutively recruited patients with acute ICH who were hospitalized in a tertiary referral teaching hospital between January 2015 and December 2020. The medication history regarding non‐aspirin NSAID use was retrospectively reviewed. Approval for this study was obtained from the Research Ethics Committee of our hospital. Patients were excluded if their hospitalization or transfer to our hospital was delayed by ≥ 10 days after onset, if they were transferred to another hospital within 10 days after ICH, or if their ICH etiologies might affect outcomes, such as trauma, structural lesions, medication, or systemic diseases‐related ICH. To avoid repeat measurements, only patients with a first‐time ICH during this period were included in the analysis.

### 2.2 Clinical Information

The data collected in this study included baseline demographic information, body mass index (BMI), comorbidities, and relevant clinical and imaging features. Comorbidities included vascular risk factors and chronic diseases requiring the use of NSAIDs. Clinical severity was assessed using the baseline National Institute of Health Stroke Scale (NIHSS) score, with an increase of at least 2 points from the baseline defined as evolution. An initial brain computed tomography (CT) scan was performed after stroke onset, which was used to diagnose ICH and assess the volume and location of the hematoma as well as the presence of intraventricular hemorrhage (IVH). The volume of hematoma was calculated using the ABC/2 method [14]. For hematomas located atypically for hypertensive angiopathy (HA) or in a typical location but without a history of hypertension, CT angiography or magnetic resonance imaging was routinely performed to investigate secondary causes. The ICH score was graded based on the Glasgow Coma Scale score, age, infratentorial origin, and volume of hematoma, as well as the presence of IVH [15]. ICH etiologies were classified using the SMASH‐U method [16]. Given the association between the initial blood pressure and ICH outcomes [17], the former was recorded as a clinical variable. Laboratory data potentially associated with ICH or non‐aspirin NSAID use were also retrieved, including hemoglobin levels, white blood cell count, platelet count, international normalized ratio, and lipid profiles. Outcomes were assessed using the modified Rankin Scale (mRS) score and mortality. The mRS score was assessed at 3 months through telephone interviews, and a good functional outcome was defined as an mRS score of 0–2. Mortality was recorded within 1 year and until the last follow‐up. The 1‐year and long‐term mortality data in the stroke registry were obtained by combining the annual records from the National Cause of Death Databank with the registration data.

### 2.3 Non‐Aspirin NSAID Use

The administration history of non‐aspirin NSAIDs before or after ICH was retrieved from the medication records in our hospital and in the National Health Insurance, and the latter covered the health insurance of almost the entire Taiwanese population [18]. Patients who regularly used one kind of NSAIDs, except aspirin, for at least three continuous days were defined as non‐aspirin NSAID users. Patients using these drugs within 1 week before ICH were classified as pre‐ICH users, and the duration was determined as the half‐lives of these medications were between 2 and 33 h [19]. Those using non‐aspirin NSAIDs within 4 weeks after ICH were defined as post‐ICH users, and the duration was defined to cover the potential period of obvious perihematomal edema [20]. The detailed history of using non‐aspirin NSAIDs included their indications, usage periods, intervals between usage periods and stroke onset, and dosages. Non‐aspirin NSAIDs administered in this study included celecoxib, etoricoxib, meloxicam, naproxen, acemetacin, diclofenac, ketorolac, and indomethacin.

### 2.4 Statistical Analysis

Continuous variables were presented as mean ± standard deviation and were compared between users and non‐users of non‐aspirin NSAIDs using either Student's *t*‐test or Mann–Whitney *U* test, depending on the normality of data distribution. The duration of non‐aspirin NSAID use and the interval between the usage period and ICH onset were compared across different indications using the Kruskal–Wallis rank test, respectively. Categorical data are shown as numbers (percentages) and were compared using the chi‐square test. Significant clinical variables associated with good functional outcomes or mortality were identified using multivariate logistic regression models, with reciprocal adjustments that included all significant variables from the univariate logistic regression models. These significant clinical variables were then used as adjusted covariates in multivariate logistic regression models to analyze the associations between non‐aspirin NSAID use and clinical outcomes. Univariate and multivariate Cox proportional hazards models were used to analyze the influence of using these medications on long‐term mortality. Long‐term Kaplan–Meier survival curves were compared between post‐ICH non‐aspirin NSAID users and non‐users using the log‐rank test. Furthermore, subgroup analyses were performed to explore the influence of clinical variables on the associations between non‐aspirin NSAID use and outcomes, including hematoma characteristics (hematoma volume and location) and clinical characteristics (sex, comorbidities, ICH subtype, and operation status), in which *p* values for interaction were calculated for all subgroups. In all these statistical analyses, *p* values less than 0.05 were considered statistically significant.

## 3 Results

**FIGURE 1:**
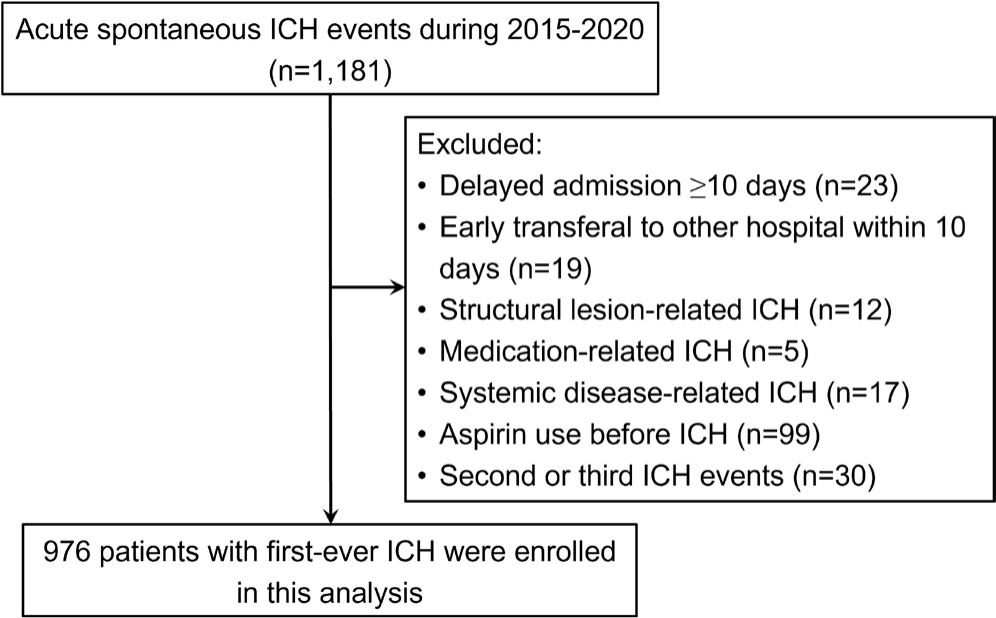
Flow chart of patient enrollment of this study.

A total of 976 patients with acute ICH were enrolled after excluding 205 (Figure 1). As outlined in baseline characteristics (Table S1), 20 patients (2.0%) were identified as non‐aspirin NSAID users prior to ICH. They were characterized by older age, higher prevalence of hyperlipidemia and osteoarthritis, and lower hemoglobin and triglyceride levels at admission (all *p* < 0.05). Post‐ICH users of non‐aspirin NSAIDs included 146 patients (15.0%), who were younger, had a lower prevalence of prior stroke, higher prevalences of osteoarthritis and gouty arthritis, lower initial NIHSS scores, higher diastolic blood pressure at admission, and were more likely to undergo surgery for ICH (all *p* < 0.05).

The usage profiles of non‐aspirin NSAIDs before and after ICH are presented in Tables S2 and S3. The medications used before ICH were mostly prescribed for chronic arthritis (osteoarthritis or rheumatoid arthritis in 60.0% of cases). In post‐ICH users, the most common indication was ICH (37.0%), followed by gouty arthritis (20.0%). Fever accounted for 15.8% of these users and was mostly attributed to ICH‐induced central fever. The interval between stroke onset and the first dose varied significantly across different indications in post‐ICH users (*p* < 0.001). Similarly, the interval between stroke onset and the last dose in pre‐ICH users showed significant variability among different indications (*p* = 0.036). However, the duration of non‐aspirin NSAID use was not variable across different indications in pre‐ or post‐ICH users.

**FIGURE 2:**
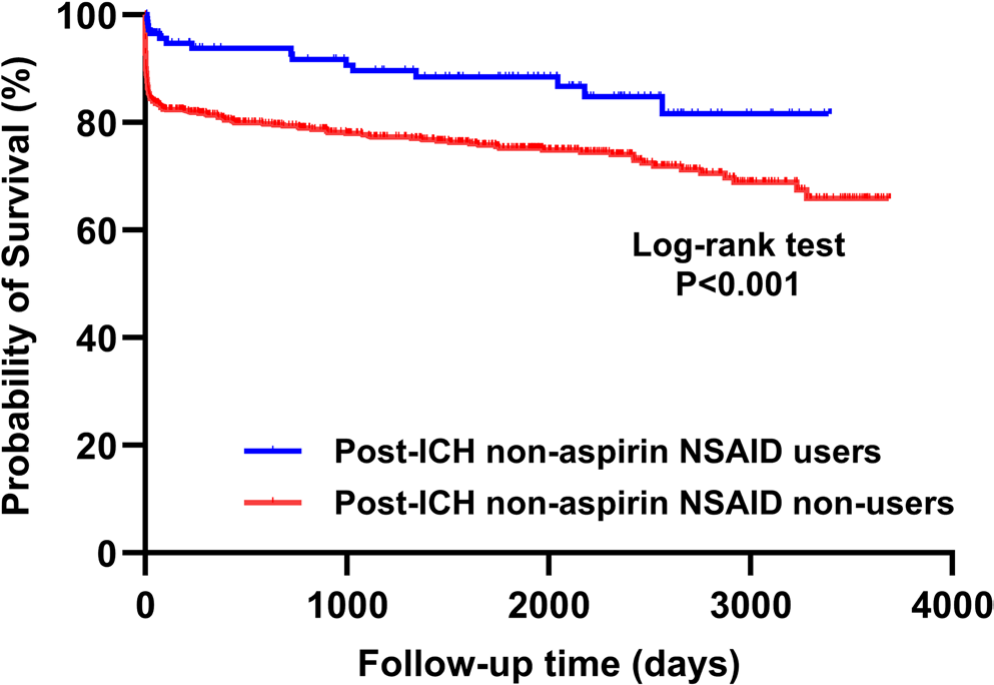
Long‐term survival based on the post‐ICH use of non‐aspirin nonsteroidal anti‐inflammatory drugs. Kaplan–Meier survival analysis showed that users of non‐aspirin nonsteroidal anti‐inflammatory drugs after ICH had a better long‐term survival compared to non‐users (log‐rank test, *p* < 0.001).

In patients with acute ICH, 322 (33.0%) achieved good functional outcomes at 3 months, 159 (16.3%) died within 1 year, and 207 (21.2%) died during the long‐term follow‐up period, with a mean duration of 1145 days (range 0–3693 days). Multivariate logistic regression analyses were performed to identify significant clinical variables associated with clinical outcomes after ICH. Following reciprocal adjustment for significant variables identified from the univariate logistic regression models, we found that younger age and lower NIHSS and ICH scores remained significantly associated with good functional outcomes at 3 months (Table S4), whereas evolution history and coronary artery disease were independently associated with an increased 1‐year mortality risk (Table S5). We also found that post‐ICH use of non‐aspirin NSAIDs was associated with a reduced 1‐year mortality risk (adjusted odds ratio [aOR] 0.30, 95% confidence interval [CI] 0.14–0.62, *p* = 0.001; Table 1) and long‐term mortality risk (adjusted hazard ratio [aHR] 0.56, 95% CI 0.32–0.98, *p* = 0.042; Table 2). In the survival curve analysis, post‐ICH non‐aspirin NSAID users had significantly better long‐term survival compared to non‐users (*p* < 0.001 for log‐rank test; Figure 2), and these two lines were separated within the first 20 days. However, the use of these medications after ICH was not associated with good functional outcomes (Table 3). Additionally, the use either before or after ICH was associated with a reduced 1‐year mortality risk (aOR 0.37, 95% CI 0.19–0.71, *p* = 0.003), but did not influence the long‐term mortality risk. In contrast, the pre‐ICH use of these medications was not linked to any of these outcomes. Regarding the usage profiles of non‐aspirin NSAIDs, the usage duration and interval between usage and stroke onset were not associated with any of these outcomes.

**TABLE 1:** Associations of using non‐aspirin nonsteroidal anti‐inflammatory drugs with mortality within 1 year.

Usage timing of non‐aspirin NSAIDs	Users *N* (%)	Non‐users *N* (%)	Crude OR (95% CI)	*p*	Adjusted OR (95% CI)^a^	*p* ^a^
Pre‐ or post‐ICH	12 (7.6%)	147 (17.9%)	0.38 (0.20, 0.70)	0.002*	0.37 (0.19, 0.71)	0.003*
Pre‐ICH	4 (20.0%)	155 (16.2%)	1.29 (0.43, 3.92)	0.651	1.79 (0.48, 6.73)	0.387
Post‐ICH	9 (6.2%)	150 (18.1%)	0.30 (0.15, 0.60)	0.001*	0.30 (0.14, 0.62)	0.001*

Abbreviations: CI, confidence interval; ICH, intracerebral hemorrhage; NSAIDs, nonsteroidal anti‐inflammatory drugs; OR, odds ratio.

^a^ Adjusted for evolution and coronary artery disease.

* *p* < 0.05.

**TABLE 2:** Associations of using non‐aspirin nonsteroidal anti‐inflammatory drugs with long‐term mortality.

Usage timing of non‐aspirin NSAIDs	Users *N* (%)	Non‐users *N* (%)	Crude HR (95% CI)	*p*	Adjusted HR (95% CI)^a^	*p* ^a^
Pre‐ or post‐ICH	20 (12.7%)	187 (22.8%)	0.53 (0.34, 0.85)	0.008*	0.62 (0.38, 1.01)	0.054
Pre‐ICH	5 (25.0%)	202 (21.1%)	1.19 (0.49, 2.88)	0.706	0.88 (0.32, 2.39)	0.795
Post‐ICH	16 (11.0%)	191 (23.0%)	0.45 (0.27, 0.75)	0.002*	0.56 (0.32, 0.98)	0.042*

Abbreviations: CI, confidence interval; HR, hazard ratio; ICH, intracerebral hemorrhage; NSAIDs, nonsteroidal anti‐inflammatory drugs.

^a^ Adjusted for age, NIHSS score, evolution, coronary artery disease, and cancer.

* *p* < 0.05.

**TABLE 3:** Associations of using non‐aspirin nonsteroidal anti‐inflammatory drugs with good functional outcome at 3 months.

Usage timing of non‐aspirin NSAIDs	Users *N* (%)	Non‐users *N* (%)	Crude OR (95% CI)	*p*	Adjusted OR (95% CI)^a^	*p* ^a^
Pre‐ or post‐ICH	57 (36.3%)	265 (32.4%)	1.19 (0.83, 1.70)	0.335	1.05 (0.63, 1.73)	0.860
Pre‐ICH	8 (40.0%)	314 (32.8%)	1.36 (0.55, 3.37)	0.502	1.76 (0.39, 7.95)	0.461
Post‐ICH	54 (37.0%)	268 (32.3%)	1.23 (0.85, 1.78)	0.266	0.98 (0.59, 1.63)	0.940

Abbreviations: CI, confidence interval; ICH, intracerebral hemorrhage; NSAIDs, nonsteroidal anti‐inflammatory drugs; OR, odds ratio.

^a^ Adjusted for age and NIHSS score.

**FIGURE 3:**
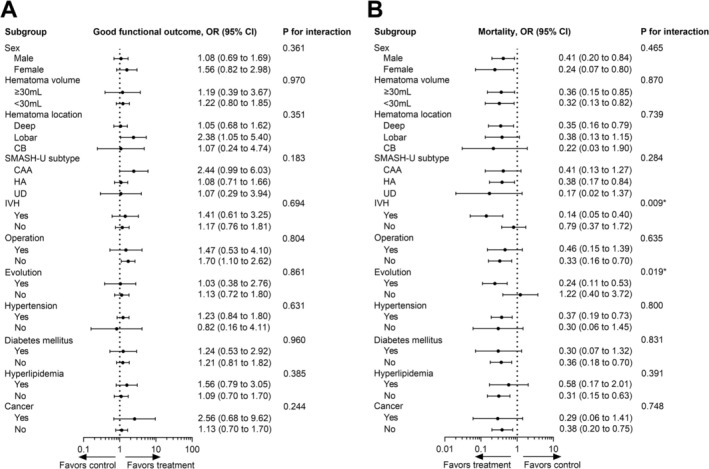
Subgroup analysis for associations between post‐ICH use of non‐aspirin nonsteroidal anti‐inflammatory drugs and ICH outcomes. Analysis of the influence of these variables in the associations of post‐ICH use of these drugs with (A) good functional outcome at 3 months and (B) mortality within 1 year.

**FIGURE 4:**
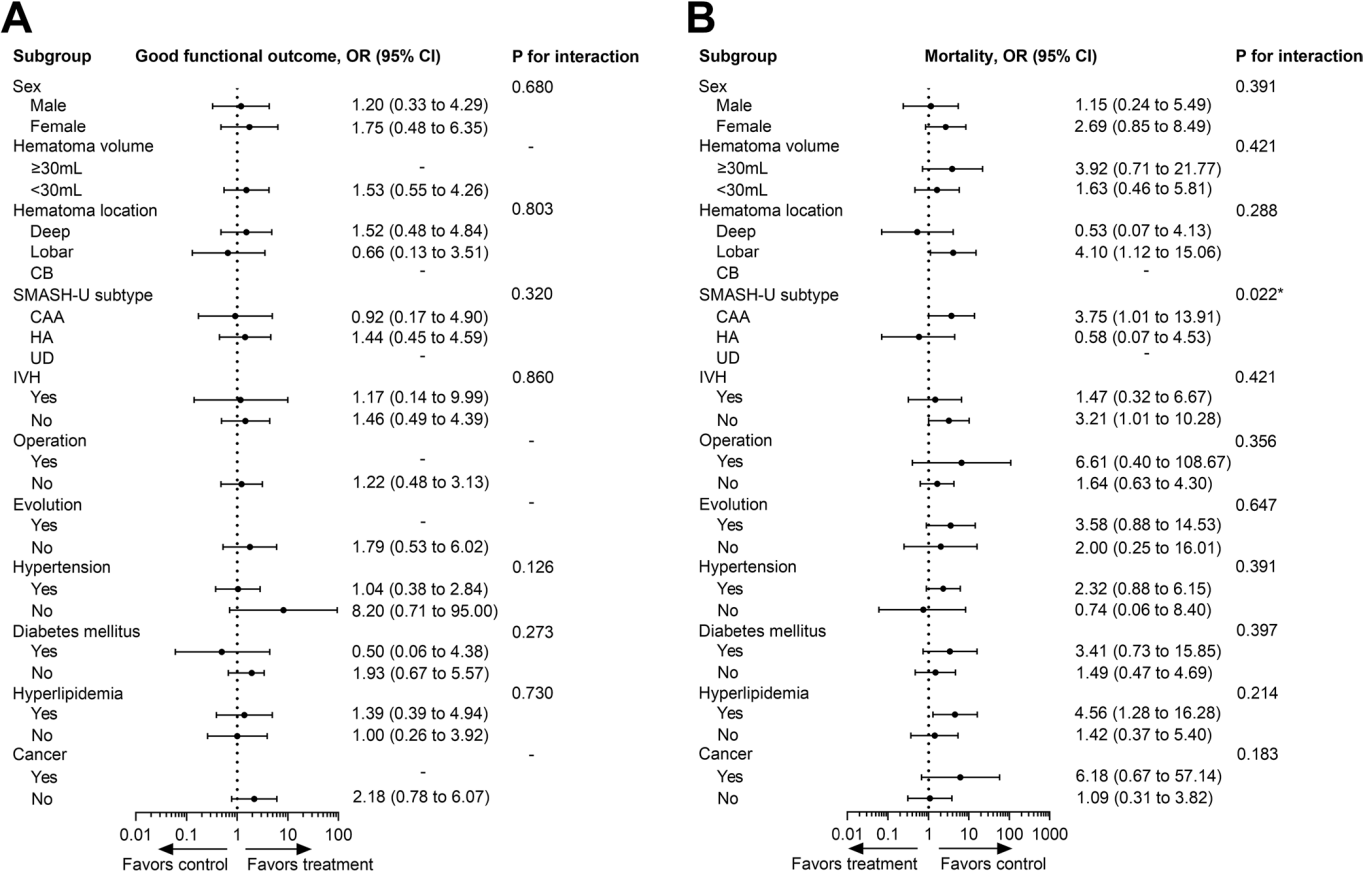
Subgroup analysis for associations between pre‐ICH use of non‐aspirin nonsteroidal anti‐inflammatory drugs and ICH outcomes. Analysis of the influence of these variables in the associations of pre‐ICH use of these drugs with (A) good functional outcome at 3 months and (B) mortality within 1 year.

Subgroup analyses were conducted to explore the influence of clinical features or hematoma characteristics on the effects of non‐aspirin NSAIDs on ICH outcomes. We found that post‐ICH use of these drugs was probably associated with good functional outcomes in the subgroup of lobar hemorrhage (OR 2.38, 95% CI 1.05–5.40) and in patients who did not undergo surgery (OR 1.70, 95% CI 1.10–2.62) (Figure 3A). Consistent with the findings in the overall population, the post‐ICH use of these drugs was associated with reduced mortality across numerous subgroups, in which patients with IVH or evolution had significant benefit in reducing mortality by post‐ICH non‐aspirin NSAIDs use (P values for interaction < 0.05) (Figure 3B). In contrast, pre‐ICH use of non‐aspirin NSAIDs was not associated with good functional outcomes in any subgroup. Instead, it was potentially associated with increased mortality in the subgroups with (1) lobar hemorrhage (OR 4.10, 95% CI 1.12–15.06), (2) cerebral amyloid angiopathy ([CAA], OR 3.75, 95% CI 1.01–13.91), (3) no IVH (OR 3.21, 95% CI 1.01–10.28), and (4) hyperlipidemia (OR 4.56, 95% CI 1.28–16.28), in which patients with CAA subtype had significant higher mortality risk by pre‐ICH non‐aspirin NSAIDs use among SMASH‐U subtypes (P values for interaction = 0.022) (Figure 4).

The impacts of surgical procedures on the outcomes in patients receiving non‐aspirin NSAIDs were investigated (Table S6). The analysis revealed that the outcomes did not vary among different surgical procedures in the patients taking these medications.

## 4 Discussion

Perihematomal edema, which is mainly mediated by inflammation, amplifies the mass effect of hematoma and potentially influences ICH outcomes. Celecoxib, a non‐aspirin NSAID, reduces perihematomal edema and mortality in patients with ICH [6, 7, 8, 9]. However, evidence is lacking regarding the effects of post‐ICH use of non‐aspirin NSAIDs on ICH outcomes and whether the pre‐ICH use of these drugs affects ICH characteristics and mortality. This study found that post‐ICH use of non‐aspirin NSAIDs was associated with reduced 1‐year and long‐term mortality in patients with ICH. Although no association was observed between post‐ICH use of these drugs and good functional outcomes in the overall population, the use of these drugs in patients with lobar hemorrhage or those without undergoing surgery might increase the likelihood of achieving good functional outcomes. Conversely, pre‐ICH use of these drugs was not associated with mortality or functional outcomes in the overall population but might be associated with increased mortality in subgroups with lobar hemorrhage, CAA, hyperlipidemia, or absence of IVH.

The effect of celecoxib in promoting functional recovery from ICH has been observed in rat models of ICH, rather than in patients with ICH [6, 7]. The small sample size of that human study might underpower the drug effect on functional outcomes [7]. Furthermore, a case report suggests that using celecoxib in patients with thalamic hematoma alleviates the neurological symptoms caused by the perihematomal edema [21]. While celecoxib offers promise, the effect of non‐aspirin NSAIDs, which share a similar pharmacological mechanism in ameliorating inflammatory reactions, on ICH outcomes remains unclear. Our study showed that the post‐ICH use of non‐aspirin NSAIDs was not associated with good functional outcomes in the overall population, indicating that perihematomal edema might not be the major contributor to the functional outcomes of ICH, or that the reduction of perihematomal edema by these drugs might not be adequate to promote functional recovery. However, the use of non‐aspirin NSAIDs after ICH may promote good functional outcomes in specific subgroups, such as those with non‐operated or lobar hematomas. Given that larger hematomas typically correlate with more extensive perihematomal edema [22], non‐operated hematomas may induce more significant perihematomal edema compared to operated ones. Thus, potentially explaining the observed benefits, the effects of non‐aspirin NSAIDs on functional outcomes could be present in non‐operated patients. We also revealed that patients with lobar hemorrhages might benefit from the post‐ICH use of non‐aspirin NSAIDs to improve functional outcomes. Lobar hematomas, compared to deep hematomas, usually exhibit less severe damage to motor pathways, which potentially contributes to better functional outcomes when the perihematomal edema is reduced with non‐aspirin NSAIDs [23].

A recent study reported that using celecoxib within 5 days of ICH reduced the 1‐year mortality risk [9]. Regarding that perihematomal edema usually expands gradually until 3 weeks after ICH [22, 24], our study defined a post‐ICH usage period of 4 weeks to cover the stage of potentially severe perihematomal edema, which revealed the beneficial effects of non‐aspirin NSAIDs on mortality reduction in ICH, not only within 1 year but also in the long‐term follow‐up. In addition, we identified subgroups in which these drugs were beneficial for reducing mortality, such as deep hematomas, non‐operated hematomas, hematomas accompanied by IVH, and hematomas with evolution. Perihematomal edema has been associated with mortality in deep hematomas [25]. Thus, it is reasonable to infer that the use of non‐aspirin NSAIDs, which reduce perihematomal edema, probably improves survival in patients with deep hematomas. In addition, IVH is an important determinant of mortality and coma in ICH patients because of the development of hydrocephalus and the induction of inflammation [26]. Removal of IVH improves consciousness, with reduced tissue inflammation in animal studies, and reduced mortality in patients [27, 28, 29]. Using non‐aspirin NSAIDs in patients with IVH may reduce inflammatory reactions and the overall mass effect of hematoma combined with perihematomal edema. Regarding surgery, hematoma evacuation reduces mortality by dramatically decreasing hematoma volume and the perihematomal edema [1]. In contrast, perihematomal edema of non‐operated hematomas was generally obvious; thus, the effect of non‐aspirin NSAIDs on reducing perihematomal edema could be significant. Our findings reveal that non‐aspirin NSAIDs may be a simple way to reduce mortality in patients with ICH.

The present study showed that the pre‐ICH use of non‐aspirin NSAIDs was not associated with functional impairment or mortality in the overall population, which was in line with a previous report [12]. However, within the lobar hemorrhage and CAA subgroups, the use of these drugs before ICH was probably associated with an increased mortality risk. CAA, characterized by lobar hemorrhage, is a chronic vasculopathy caused by the deposition of amyloid in the cortical and meningeal blood vessels [30]. A previous human neuropathology study has shown that the heavy use of nonselective NSAIDs was not associated with the occurrence of CAA [31], and that the use of flurbiprofen, a non‐aspirin NSAID, did not affect vascular amyloid deposition or microhemorrhage in amyloid‐beta precursor protein transgenic mice [32]. Further studies are needed to understand the impact of non‐aspirin NSAIDs on the outcomes of CAA‐related ICH.

Our study has several strengths, including a large population with long‐term follow‐up and adjustments for covariates in the statistical analyses. However, it also has some limitations. First, patients who received palliative care for ICH usually did not receive NSAIDs, introducing potential confounding in the analysis of drug effects. Second, the history of pre‐ICH non‐aspirin NSAID use via over‐the‐counter purchases might have been missed; however, its influence on the study is likely minimal because of the lower cost of prescribed medication than over‐the‐counter purchases, along with the consistent documentation in admission records. Third, owing to the lack of evaluation of genetic profiles that influence NSAIDs pharmacokinetics, these findings may not be generalizable to different populations, and this study could not evaluate the biological outcomes. Fourth, the lack of patient blinding might have caused bias in this observational study. Lastly, this study lacked validation in a second population. Further randomized controlled trials are essential to validate our findings.

## 5 Conclusions

This study elucidated the effects of non‐aspirin NSAIDs on ICH patients. The post‐ICH use of these medications was linked to a reduction in both 1‐year and long‐term mortality risks and showed good functional outcomes in patients with lobar hemorrhage or those without operation. Pre‐ICH use of these drugs was potentially linked to increased mortality in subgroups with lobar hemorrhage, CAA, hyperlipidemia, or no IVH. Future studies are warranted to identify a specific non‐aspirin NSAID regimen that predominantly benefits patients with ICH.

## Author Contributions

S.‐J.Y., S.‐C.H, and J.‐S.J. designed the study. J.‐S.J. and S.‐J.Y. collected the data. S.‐J.Y. analyzed the data and wrote the initial draft of the manuscript. All authors contributed to editing the manuscript by providing critical feedback and approved the final version.

## Conflicts of Interest

The authors declare no conflicts of interest.

## Supporting information


**Table S1:** Baseline characteristics according to the use of non‐aspirin nonsteroidal anti‐inflammatory drugs.
**Table S2:** The indications and timings of using non‐aspirin nonsteroidal anti‐inflammatory drugs before acute intracerebral hemorrhage.
**Table S3:** The indications and timings of using non‐aspirin nonsteroidal anti‐inflammatory drugs after acute intracerebral hemorrhage.
**Table S4:** Significant clinical variables associated with good functional outcomes at 3 months.
**Table S5:** Significant clinical variables associated with mortality within 1 year.
**Table S6:** Outcomes according to surgery types in patients using non‐aspirin nonsteroidal anti‐inflammatory drugs and undergoing surgeries.

## Data Availability

The data that support the findings of this study are available from the corresponding author upon reasonable request.
