# Supplementary material for: Effects of Non‐Aspirin Nonsteroidal Anti‐Inflammatory Drugs on Acute Intracerebral Hemorrhage
Source: Ann Clin Transl Neurol. 2025 Aug 15;12(11):2328–34. doi: 10.1002/acn3.70163 (PMC12623835; doi:10.1002/acn3.70163)
Supplement: Supplementary file 1 — Table S1: Baseline characteristics according to the use of non‐aspirin nonsteroidal anti‐inflammatory drugs. Table S2: The indications and timings of using non‐aspirin nonsteroidal anti‐inflammatory drugs before acute intracerebral hemorrhage. Table S3: The indications and timings of using non‐aspirin nonsteroidal anti‐inflammatory drugs after acute intracerebral hemorrhage. Table S4: Significant clinical variables associated with good functional outcomes at 3 months. Table S5: Significant clinical variables associated with mortality within 1 year. Table S6: Outcomes according to surgery types in patients using non‐aspirin nonsteroidal anti‐inflammatory drugs and undergoing surgeries. [file ACN3-12-2328-s001.docx]

Table S1. Baseline characteristics according to the use of non-aspirin nonsteroidal anti-inflammatory drugs

|  | Usage in pre-ICH stage | | | Usage in post-ICH stage | | |
| --- | --- | --- | --- | --- | --- | --- |
|  | Users  (n=20) | Non-users  (n=956) | P-value | Users  (n=146) | Non-users  (n=830) | P-value |
| Age (years) | 67.7 ± 11.0 | 62.2 ± 14.9 | 0.039* | 59.9 ± 14.3 | 62.7 ± 15.0 | 0.034* |
| Sex, male | 10 (50.0) | 615 (64.3) | 0.238 | 99 (67.8) | 526 (63.4) | 0.350 |
| BMI (kg/m^2^) | 23.7 ± 4.6 | 25.0 ± 4.8 (n=807) | 0.210 | 25.3 ± 4.5 (n=126) | 24.9 ± 4.9 (n=701) | 0.358 |
| Comorbidities |  |  |  |  |  |  |
| Hypertension | 17 (85.0) | 854 (89.3) | 0.467 | 135 (92.5) | 736 (88.7) | 0.194 |
| Diabetes mellitus | 6 (30.0) | 214 (22.4) | 0.420 | 28 (19.2) | 192 (23.1) | 0.334 |
| Hyperlipidemia | 10 (50.0) | 237 (24.8) | 0.017* | 41 (28.1) | 206 (24.8) | 0.410 |
| Prior stroke | 1 (5.0) | 132 (13.8) | 0.504 | 12 (8.2) | 121 (14.6) | 0.037* |
| Coronary artery disease | 0 (0) | 49 (5.1) | 0.619 | 6 (4.1) | 43 (5.2) | 0.685 |
| Smoking | 5 (25.0) | 260 (27.2) | 1.000 | 44 (30.1) | 221 (26.6) | 0.419 |
| Alcohol use | 2 (10.0) | 159 (16.6) | 0.556 | 26 (17.8) | 135 (16.3) | 0.630 |
| Osteoarthritis | 13 (65.0) | 76 (7.9) | <0.001* | 21 (14.4) | 68 (8.2) | 0.028* |
| Rheumatoid arthritis | 1 (5.0) | 2 (0.2) | 0.060 | 2 (1.4) | 1 (0.1) | 0.060 |
| Gouty arthritis | 3 (15.0) | 76 (7.9) | 0.216 | 37 (25.3) | 42 (5.1) | <0.001* |
| Hematoma volume (mL) | 22.3 ± 27.1 | 29.0 ± 37.0 | 0.303 | 25.7 ± 29.3 | 29.5 ± 38.0 | 0.176 |
| Baseline NIHSS score | 13.1 ± 12.0 (n=17) | 15.5 ± 11.3 (n=923) | 0.413 | 12.6 ± 9.2 (n=139) | 16.0 ± 11.6 (n=801) | <0.001* |
| Evolution | 8 (40.0) | 431 (45.1) | 0.821 | 58 (39.7) | 381 (45.9) | 0.177 |
| Hematoma location |  |  | 0.274 |  |  | 0.744 |
| Deep region | 12 (60.0) | 704 (73.6) |  | 109 (74.7) | 607 (73.1) |  |
| Lobar region | 7 (35.0) | 194 (20.3) |  | 27 (18.5) | 174 (21.0) |  |
| Cerebellum | 1 (5.0) | 58 (6.1) |  | 10 (6.8) | 49 (5.9) |  |
| Intraventricular hemorrhage | 7 (35.0) | 346 (36.2) | 1.000 | 50 (34.2) | 303 (36.5) | 0.641 |
| ICH score, median (IQR) | 1 (2) | 1 (2) | 0.921 | 1 (2) | 1 (2) | 0.438 |
| SMASH-U etiology |  |  | 0.056 |  |  | 0.491 |
| Cerebral amyloid angiopathy | 7 (35.0) | 148 (15.5) |  | 23 (15.8) | 132 (15.9) |  |
| Hypertensive angiopathy | 12 (60.0) | 709 (74.2) |  | 112 (76.7) | 609 (73.4) |  |
| Undetermined etiology | 1 (5.0) | 99 (10.4) |  | 11 (7.5) | 89 (10.7) |  |
| BP at admission |  |  |  |  |  |  |
| Systolic BP (mmHg) | 175.2 ± 31.0 | 179.2 ± 38.0 | 0.576 | 181.3 ± 34.0 | 178.7 ± 38.5 | 0.416 |
| Diastolic BP (mmHg) | 95.8 ± 22.7 | 100.1 ± 22.7 | 0.409 | 104.4 ± 21.0 | 99.3 ± 22.9 | 0.009* |
| Pulse pressure (mmHg) | 79.4 ± 18.8 | 78.2 ± 28.5 | 0.786 | 76.6 ± 28.3 | 78.5 ± 28.3 | 0.441 |
| Laboratory data on admission |  |  |  |  |  |  |
| Hemoglobin (g/dL) | 13.1 ± 2.0 | 14.1 ± 2.2 | 0.036* | 14.3 ± 1.9 | 14.1 ± 2.2 | 0.251 |
| WBC count (K/μL) | 9.6 ± 4.3 | 10.1 ± 5.8 | 0.590 | 10.1 ± 4.3 | 10.1 ± 6.0 | 0.879 |
| Platelet count (K/μL) | 200.1 ± 75.6 | 228.7 ± 72.2 | 0.110 | 224.3 ± 68.5 | 228.8 ± 73.0 | 0.470 |
| INR | 1.0 ± 0.1 | 1.0 ± 0.1 (n=934) | 0.757 | 1.0 ± 0.1 (n=145) | 1.0 ± 0.1  (n=809) | 0.803 |
| LDL (mg/dL) | 106.0 ± 47.4 (n=14) | 108.0 ± 33.3 (n=565) | 0.877 | 108.2 ± 33.8 (n=88) | 107.9 ± 33.6 (n=491) | 0.939 |
| TG (mg/dL) | 101.5 ± 31.8 (n=15) | 124.2 ± 84.2 (n=579) | 0.019* | 124.4 ± 73.1 (n=89) | 123.5 ± 85.0 (n=505) | 0.919 |
| HDL (mg/dL) | 46.1 ± 16.1 (n=14) | 45.8 ± 12.5 (n=544) | 0.938 | 43.8 ± 12.8 (n=83) | 46.2 ± 12.5 (n=475) | 0.117 |
| T-CHO (mg/dL) | 168.7 ± 65.0 (n=15) | 176.3 ± 37.9 (n=589) | 0.658 | 174.6 ± 41.5 (n=91) | 176.4 ± 38.3 (n=513) | 0.698 |
| Operation | 2 (10.0) | 210 (22.0) | 0.276 | 52 (35.6) | 160 (19.3) | <0.001* |
| Hematoma evacuation | 1 (5.0) | 172 (18.0) |  | 42 (28.8) | 131 (15.8) |  |
| Extraventricular drainage | 1 (5.0) | 38 (4.0) |  | 10 (6.8) | 29 (3.5) |  |

*, P<0.05

Data are shown as number (%), mean ± standard deviation or median (interquartile range).

Abbreviations: BMI, body mass index; BP, blood pressure; HDL, high-density lipoprotein; ICH, intracerebral hemorrhage; INR, international normalized ratio; IQR, interquartile range; LDL, low-density lipoprotein; NIHSS, National Institutes of Health Stroke Scale; T-CHO, total cholesterol; TG, Triglyceride; WBC, white blood cell

Table S2. The indications and timings of using non-aspirin nonsteroidal anti-inflammatory drugs before acute intracerebral hemorrhage

| Indication | Number of users  (n=20) | Interval between stroke onset and the last dose (days) * | Usage duration within 1 week before stroke onset (days) † |
| --- | --- | --- | --- |
| Chronic arthritis | 12 (60.0%) | 0.1 ± 0.3 | 6.9 ± 0.3 |
| Gouty arthritis | 2 (10.0%) | 0 ± 0 | 7.0 ± 0 |
| Soft tissue pain | 4 (20.0%) | 0 ± 0 | 6.8 ± 0.5 |
| Cancer pain | 1 (5.0%) | 4.0 | 3.0 |
| Unknown | 1 (5.0%) | 0 | 7.0 |

Chronic arthritis included osteoarthritis and rheumatoid arthritis.

* Comparison of intervals among different indications: P = 0.036

† Comparison of usage durations among different indications: P = 0.097

Table S3. The indications and timings of using non-aspirin nonsteroidal anti-inflammatory drugs after acute intracerebral hemorrhage

| Indication | Number of users  (n=146) | Interval between stroke onset and the first dose (days) * | Usage duration within 30 days after stroke onset (days) † |
| --- | --- | --- | --- |
| Intracerebral hemorrhage | 54 (37.0%) | 6.1 ± 4.6 | 9.4 ± 6.4 |
| Fever | 23 (15.8%) | 9.6 ± 4.9 | 7.3 ± 4.8 |
| Gouty arthritis | 29 (20.0%) | 9.3 ± 5.9 | 7.7 ± 6.2 |
| Chronic arthritis† | 12 (8.2%) | 17.9 ± 7.6 | 8.9 ± 5.1 |
| Soft tissue pain | 26 (17.8%) | 16.4 ± 7.9 | 6.5 ± 4.8 |
| Fracture | 2 (1.4%) | 16.0 ± 18.4 | 3.5 ± 2.1 |

Fever was caused by intracerebral hemorrhage-induced central fever or infection. Chronic arthritis included osteoarthritis and rheumatoid arthritis.

* Comparison of intervals among different indications: P <0.001

† Comparison of usage durations among different indications: P = 0.069

Table S4. Significant clinical variables associated with good functional outcomes at 3 months

|  | Crude OR  (95% CI) | P value | Adjusted OR  (95% CI) † | P value |
| --- | --- | --- | --- | --- |
| Age (years) | 0.96 (0.95, 0.97) | <0.001* | 0.95 (0.93, 0.97) | <0.001* |
| Sex, male | 1.44 (1.08, 1.92) | 0.012* | 1.09 (0.58, 2.04) | 0.797 |
| BMI (kg/m^2^) | 1.07 (1.04, 1.10) | <0.001* | 0.99 (0.93, 1.07) | 0.883 |
| Hypertension | 1.98 (1.21, 3.24) | 0.006* | 1.49 (0.36, 6.19) | 0.582 |
| Dyslipidemia | 1.70 (1.27, 2.30) | <0.001* | 0.73 (0.38, 1.40) | 0.347 |
| Prior stroke | 0.47 (0.30, 0.74) | 0.001* | 0.61 (0.26, 1.40) | 0.242 |
| Coronary artery disease | 0.38 (0.18, 0.82) | 0.014* | 0.46 (0.10, 2.08) | 0.311 |
| Cancer | 0.47 (0.29, 0.76) | 0.002* | 1.47 (0.51, 4.26) | 0.475 |
| Osteoarthritis | 0.49 (0.28, 0.83) | 0.008* | 0.47 (0.19, 1.18) | 0.108 |
| Gouty arthritis | 2.00 (1.26. 3.18) | 0.003* | 1.11 (0.47, 2.60) | 0.812 |
| Hematoma volume (mL) | 0.95 (0.94, 0.96) | <0.001* | 1.01 (0.98, 1.04) | 0.485 |
| NIHSS score | 0.79 (0.77, 0.82) | <0.001* | 0.79 (0.74, 0.83) | <0.001* |
| Evolution | 0.08 (0.06, 0.12) | <0.001* | 0.50 (0.24, 1.06) | 0.070 |
| IVH | 0.18 (0.12, 0.25) | <0.001* | 1.18 (0.54, 2.61) | 0.680 |
| ICH score | 0.26 (0.22, 0.32) | <0.001* | 0.56 (0.34, 0.92) | 0.022* |
| Pulse pressure (mmHg) | 0.99 (0.99, 1.00) | 0.001* | 0.99 (0.98, 1.01) | 0.334 |
| Hemoglobin (g/dL) | 1.19 (1.11, 1.27) | <0.001* | 1.04 (0.87, 1.24) | 0.692 |
| WBC count (K/μL) | 0.92 (0.89, 0.96) | <0.001* | 0.97 (0.88, 1.07) | 0.542 |
| INR | 0.03 (0.01, 0.26) | 0.001* | 0.33 (0.00, 25.55) | 0.617 |
| LDL (mg/dL) | 1.01 (1.00, 1.01) | <0.001* | 1.01 (0.99, 1.03) | 0.310 |
| T-CHO (mg/dL) | 1.01 (1.00, 1.01) | <0.001* | 0.99 (0.97, 1.01) | 0.301 |
| TG (mg/dL) | 1.00 (1.00, 1.00) | 0.032* | 1.00 (1.00, 1.01) | 0.559 |
| Operation | 0.15 (0.09, 0.25) | <0.001* | 0.85 (0.20, 3.63) | 0.825 |
| Hematoma evacuation | 0.17 (0.10, 0.28) | <0.001* | - | - |
| EVD | 0.08 (0.02, 0.34) | 0.001* | - | - |

*, P<0.05

† Multivariate logistic regression analysis: reciprocal adjustment of all these significant variables identified in univariate logistic regression models.

Abbreviations: BMI, body mass index; EVD, external ventricular drainage; ICH, intracerebral hemorrhage; INR, international normalized ratio; IVH, intraventricular hemorrhage; LDL, low-density lipoprotein; NIHSS, National Institutes of Health Stroke Scale; OR, odds ratio; T-CHO, total cholesterol; TG, triglyceride; WBC, white blood cell

Table S5. Significant clinical variables associated with mortality within 1 year

|  | Crude OR  (95% CI) | P value | Adjusted OR  (95% CI)† | P value |
| --- | --- | --- | --- | --- |
| Age (years) | 1.03 (1.01, 1.04) | <0.001* | 1.01 (0.96, 1.07) | 0.627 |
| BMI (kg/m^2^) | 0.90 (0.86, 0.95) | <0.001* | 0.86 (0.72, 1.03) | 0.109 |
| Hypertension | 0.22 (0.14, 0.34) | <0.001* | 0.21 (6.91e-07, 62212.60) | 0.807 |
| Dyslipidemia | 0.45 (0.28, 0.71) | 0.001* | 0.34 (0.05, 2.13) | 0.246 |
| Coronary artery disease | 3.24 (1.76, 5.94) | <0.001* | 20.43 (1.56, 268.29) | 0.022* |
| Cancer | 3.49 (2.27, 5.35) | <0.001* | 4.87 (0.76, 31.17) | 0.095 |
| Gouty arthritis | 0.40 (0.17, 0.94) | 0.035* | 1.52 (0.13, 17.22) | 0.734 |
| Hematoma volume (mL) | 1.02 (1.02, 1.03) | <0.001* | 0.95 (0.89, 1.01) | 0.133 |
| NIHSS score | 1.19 (1.16, 1.22) | <0.001* | 1.09 (0.99, 1.20) | 0.080 |
| Evolution | 40.10 (18.55, 86.70) | <0.001* | 9.47 (1.75, 51.16) | 0.009* |
| ICH location (ref: deep region) |  |  |  |  |
| Lobar region | 1.95 (1.32, 2.87) | 0.001* | - | - |
| IVH | 6.36 (4.35, 9.29) | <0.001* | 1.07 (0.20, 5.62) | 0.936 |
| ICH score | 3.41 (2.84, 4.08) | <0.001* | 2.51 (0.98, 6.42) | 0.054 |
| SMASH-U etiology (ref: hypertensive angiopathy) |  |  |  |  |
| CAA | 2.43 (1.58, 3.74) | <0.001* | - | - |
| Undetermined | 4.23 (2.65, 6.74) | <0.001* | - | - |
| Diastolic BP (mmHg) | 0.99 (0.98, 1.00) | 0.002* | 0.99 (0.95, 1.02) | 0.421 |
| Hemoglobin (g/dL) | 0.76 (0.70, 0.82) | <0.001* | 0.84 (0.53, 1.34) | 0.469 |
| WBC count (K/μL) | 1.12 (1.08, 1.16) | <0.001* | 1.15 (0.96, 1.38) | 0.133 |
| Platelet count (K/μL) | 1.00 (0.99, 1.00) | 0.011* | 1.00 (0.99, 1.01) | 0.674 |
| INR | 1266.35 (143.46, 11178.52) | <0.001* | 0.25 (2.38e-06, 26665.94) | 0.815 |
| LDL (mg/dL) | 0.99 (0.98, 1.00) | 0.035* | 1.03 (0.99, 1.07) | 0.120 |
| T-CHO (mg/dL) | 0.99 (0.98, 1.00) | 0.012* | 0.98 (0.95, 1.02) | 0.290 |
| Operation (ref: no operation) | 0.63 (0.40, 0.99) | 0.046* | 0.10 (0.01, 1.42) | 0.089 |
| Hematoma evacuation | 0.55 (0.33, 0.93) | 0.026* | - | - |
| EVD | 1.03 (0.44, 2.38) | 0.948 | - | - |

*, P<0.05

†Multivariate logistic regression analysis: reciprocal adjustment of all these significant variables identified in univariate logistic regression models.

Abbreviations: BMI, body mass index; EVD, external ventricular drainage; ICH, intracerebral hemorrhage; INR, international normalized ratio; IVH, intraventricular hemorrhage; LDL, low-density lipoprotein; NIHSS, National Institutes of Health Stroke Scale; OR, odds ratio; ref, reference; WBC, white blood cell

Table S6. Outcomes according to surgery types in patients using non-aspirin nonsteroidal anti-inflammatory drugs and undergoing surgeries

|  | Good functional outcome at 3 months | | |  | Mortality within 1 year | | |
| --- | --- | --- | --- | --- | --- | --- | --- |
| Usage timing | HE | EVD | P value |  | HE | EVD | P value |
| Pre- or post-ICH | 6 (14.0%) | 0 (0%) | 0.327 |  | 3 (7.0%) | 0 (0%) | 1.000 |
| Pre-ICH | 0 (0%) | 0 (0%) | - |  | 0 (0%) | 0 (0%) | - |
| Post-ICH | 6 (14.3%) | 0 (0%) | 0.582 |  | 3 (7.1%) | 0 (0%) | 1.000 |

Abbreviations: EVD, external ventricular drainage; HE, hematoma evacuation; ICH, intracerebral hemorrhage

The numbers (percentages) showed those with these outcomes in patients undergoing specific surgeries.
